# GxxxG Motif Stabilize Ion-Channel like Pores through C_α_―H···O Interaction in Aβ (1-40)

**DOI:** 10.3390/ijms24032192

**Published:** 2023-01-22

**Authors:** Carola Rando, Giuseppe Grasso, Dibakar Sarkar, Michele Francesco Maria Sciacca, Lorena Maria Cucci, Alessia Cosentino, Giuseppe Forte, Martina Pannuzzo, Cristina Satriano, Anirban Bhunia, Carmelo La Rosa

**Affiliations:** 1 Dipartimento di Scienze Chimiche, Università degli Studi di Catania, Viale A. Doria 6, 95125 Catania, Italy; 2 Department of Biophysics Bose Institute, Unified Academic Campus, Bidhan Nagar, EN 80, Kolkata 700091, India; 3 Consiglio Nazionale delle Ricerche, Istituto di Cristallografia, 95121 Catania, Italy; 4 Dipartimento di Scienze del Farmaco e della Salute, Università degli Studi di Catania, Viale A. Doria 6, 95125 Catania, Italy; 5 Laboratory of Nanotechnology for Precision Medicine, Fondazione Istituto Italiano di Tecnologia, Via Morego 30, 16163 Genoa, Italy

**Keywords:** Aβ, ion-channel-like, pore, membrane, toxicity, FTIR-ATR, quantum mechanics, AFM, hydrogen bond

## Abstract

Aβ (1-40) can transfer from the aqueous phase to the bilayer and thus form stable ion-channel-like pores where the protein has alpha-helical conformation. The stability of the pores is due to the presence of the GXXXG motif. It has been reported that these ion-channel-like pores are stabilized by a Cα―H···O hydrogen bond that is established between a glycine of the GXXXG sequence of an alpha-helix and another amino acid of a vicinal alpha-helix. However, conflicting data are reported in the literature. Some authors have suggested that hydrogen bonding does not have a stabilizing function. Here we synthesized pentapeptides having a GXXXG motif to explore its role in pore stability. We used molecular dynamics simulations, quantum mechanics, and experimental biophysical techniques to determine whether hydrogen bonding was formed and had a stabilizing function in ion-channel-like structures. Starting from our previous molecular dynamics data, molecular quantum mechanics simulations, and ATR data showed that a stable ion-channel-like pore formed and a band centered at 2910 cm^−1^ was attributed to the interaction between Gly 7 of an alpha-helix and Asp 23 of a vicinal alpha-helix.

## 1. Introduction

Protein misfolding and assembly are complex, intertwined processes resulting in the development of a heterogeneous population of aggregates closely related to many chronic pathological conditions, including type 2 diabetes mellitus (T2DM) [1], Parkinson’s disease (PD) [2], and Alzheimer’s disease (AD) [3]. Proteins associated with these diseases are called intrinsically disordered proteins (IDPs) because they do not have a well-defined tertiary structure but explore a large conformational space ensemble [4]. IDPs can also cause membrane damage, which is crucial in the pathogenesis of protein-misfolding diseases. To develop effective drug candidates against these diseases, a comprehensive understanding of the molecular mechanism by which IDPs disrupt the membrane is imperative. Based on biophysical data, three models of the so-called amyloid cascade hypothesis that are compatible with membrane damage have been developed [5]. From a kinetic point-of-view, in the first stage, the generation of a stable transmembrane ion-channel-like pores was detected (poration mechanism), and sequentially, membrane destabilization via a “carpet model” or phospholipid removal from the bilayer by a detergent-like mechanism [6]. In addition, a molecular mechanism that describes the transfer of IDPs from the aqueous phase to the hydrocarbon core of the bilayer, called the lipid-chaperone hypothesis was recently proposed [7,8,9,10], i.e., free phospholipids in the aqueous phase (in chemical equilibrium with phospholipid bilayers) can form a lipid–protein complex having higher hydrophobicity than the bare protein, and thus being more prone to transfer into the bilayer. From an experimental standpoint, the detection of transmembrane ion-channels occurred via a fluorescent probe loaded into the lumen of LUVs, the most-used membrane mimetics. It has been reported that Aβ-induced pores were detected using a fura 2/Ca++ probe, whereas hIAPP and α-synuclein pores were detected using a carboxyfluorescein fluorescent probe. Ca++ ions cross the membrane through pores having a dimension of a few nm; however, carboxyfluorescein needs membrane pores of 10 nm in size [9]. The observation that Aβ forms tiny pores compared to hIAPP and α-synuclein can further elucidate the toxicity of IDPs. Recently, it was shown that IDP involvement in the aggregation process occurs if the intermolecular interactions exceed intramolecular forces [11]. This suggests that the neighbor-to-neighbor interactions of Aβ should be stronger with respect to each other’s IDPs. On the other hand, it has been previously suggested that the presence of the GXXXG sequence on glycophorin A and IDPs plays a significant role in stabilizing ion-channel-like pores through weak hydrogen bonds between Cα―H of an α-helix and C=O of the neighboring helix [12,13,14,15,16,17]. A recent finding about a similar behavior has also been reported for the C-terminus fragment Aβ (20-40) containing three GXXXG sequences [18]. This fragment showed toxic behavior as the full-length protein, but the mutations of Gly amino acid to Leu decreased the toxicity, and all Gly-mutated peptides became nontoxic. This behavior was attributed to the formation of the Cα―H···O=C hydrogen bond. However, contradictory data can be found in literature. Some authors suggest the central role of such a hydrogen bond in the helix–helix ion channel, estimating a value of about 3.68 kJmole^−1^ [12]; on the other hand, according to other authors, no significant stabilization can be attributed to that bond [13].

Here, we used various biophysical techniques to determine if the Cα―H···O interaction between two proximal α-helices in membrane and water was formed, considering both the full-length polypeptide and GXXXG pentapeptides units. Figure S1 shows the workflow of the experiments and related conclusions. Moreover, the influence of the sequence of the X-side chain was explored according to the hydrophobicity, charge, and hydrophilicity of amino acids.

## 2. Results

### 2.1. Molecular Dynamics and Quantum Mechanics Reveal C_α_―H···O Interaction in Aβ(1-40) Ion-Channel like Pores

**Figure 1:**
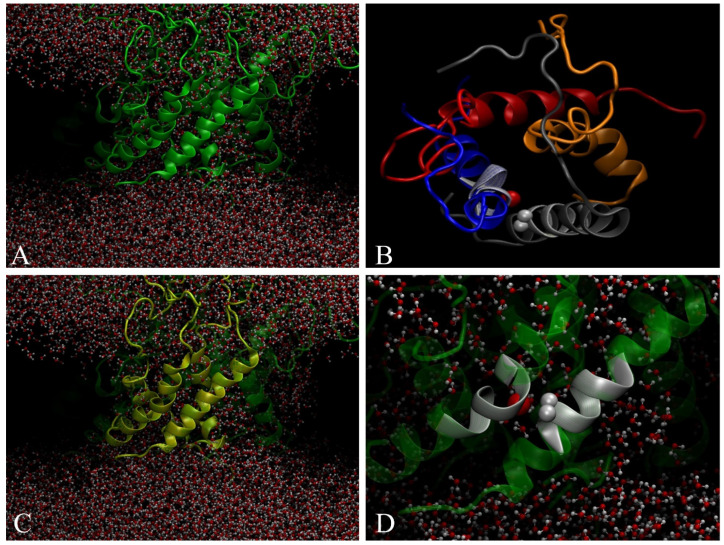
(**A**) 24-mer of Aβ (1-40) within POPC bilayer forming ion-channel-like pores where water is able to cross the bilayer. (**B**) Top view of tetramer ion-channel-like pore. (**C**) Tetramer forming structured pore. (**D**) Two crossed a-helix inside the tetramer by forming an angle of 39 ± 5 degrees. Gray spheres represent two C_α_-hydrogen of Gly and the red sphere represents C=O carbonyl oxygen.

Multi-scale molecular dynamics simulations have been performed previously by the authors to study the spontaneous aggregation of Aβ(1-40) embedded into a POPC membrane [19]. The 24 transmembrane peptides were found to self-assemble to form a truncated cone-shape aggregate. Due to the peculiar geometry of the aggregate, the membrane changed its curvature radius. It was also observed that the aggregate contained ion-channel-like pores to allow ions and water to cross the bilayer (Figure 1A). This behavior has been explained with a model called *frustrated-helix*. It has been deduced that the leading cause of the change in the bending radius is due to electrostatic repulsions of the terminal COOH and NH_2_ hydrophilic amino acids, which are exposed to the solvent. In addition, a large amount of Aβ(1-40) peptides remain in α-helix conformation; the internal organization shows dimers and trimers twisted by an angle of 39 ± 5 degrees, as shown in Figure 1. The Aβ(1-40) aggregate in membrane consists of small ion-channel-like transmembrane subunits containing four Aβ molecules as shown in Figure 1B. For clarity, just the pore is shown (top view).

It has been recently reported that the toxicity of Aβ (1-40) can be mainly related to the GXXXG pattern, which is repeated four times in the polypeptide chain on the COOH-terminal side. In particular, the G^33^XXXG^37^ sequence is primarily responsible for the neurotoxicity of Aβ (1-40) [18]. On the other hand, it has been proposed that the GXXXG sequence plays a major role in the stabilization of transmembrane proteins, forming a hydrogen bond between the Cα hydrogen of an alpha helix and the C=O of the proximal alpha helix. Fourier-transform infrared spectroscopy (FTIR) measurements estimate a value of 0.88 kcal/mole, which is 2.5–3.5 lower than the value obtained from ab-initio gas-phase calculations [12].

**Figure 2:**
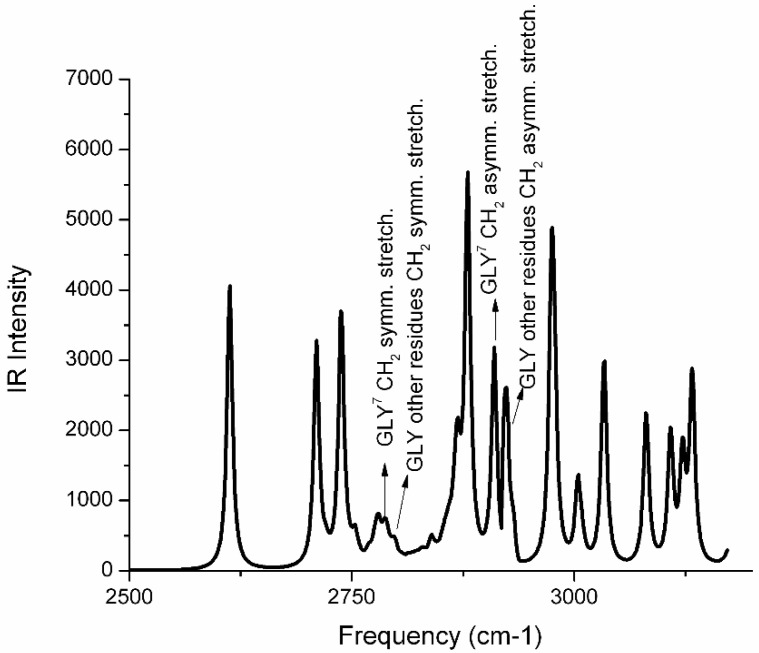
QM—simulated IR spectrum of two proximal Aβ (1-40) α-helices of a tetramer ion-channel pore as reported in Figure 1D. The amino acid sequence is reported in the Materials and Methods section. A hydrogen bond is established between the C^α^-H of Gly29 (in spectrum numbered as Gly7) and the C=O group of Asp23.

Here we performed quantum mechanics (QM) calculations to evaluate the hydrogen bond value between two proximal α-helices within an Aβ (1-40) aggregate (see Figure 1D, α-helix in gray). Our study was mainly focused on the interactions between the Hα atoms of glycine residues and carbonyl oxygen atoms spatially close to them. From a topological point-of-view, we observed an inter-chain interaction involving the residues Gly29—c2 and Asp23—c1 for which the Hα—O=C distance was 3.32 Å, and the related Wiberg bond index was 0.001. It is to note that several intra-chain interactions were also found between Gly and other residues. The calculated vibrational spectrum shows that symmetric CH_2_ stretching vibrations of Gly7 occurred at 2787 cm^−1^ and were low-shifted in the frequency of about 10 cm^−1^ compared to symmetric stretching values of methylene groups of the other Gly residues, see Figure 2. The CH_2_ asymmetric stretching modes of Gly residues increased in intensity compared to the symmetric vibrations and, similarly to the symmetric case, the value assigned to Gly7 (2910 cm^−1^) was at a lower frequency, of about 10–15 cm^−1^, as compared to the remaining Gly residues. In summary, the infrared (IR) spectrum captured different vibrational behaviors as a function of the effectiveness of the inter-chain interactions involving the glycine.

Molecular dynamics and quantum mechanics calculations suggest the formation of a lock between two proximal α-helices is mediated by a GXXXG motif. So the question is: Does the GXXXG motif have an intrinsic ability to form an intermolecular bridge regardless of the external environment, or is this a specific behavior shown only when the polypeptide containing the GXXXG motif is inserted into the membrane? To respond to this question, we designed an experiment in water and in *n*-octanol to investigate the behavior of the pentapeptide GXXXG. In addition, the sequence effect was explored. *n*-octanol was selected because we maintained the hydrophobic environment but avoided the anisotropy constraint of the membrane, and also avoided water interfering with FTIR spectra.

### 2.2. Penta-Peptide GXXXG in Water Does Not Form the C_α_―H···O Hydrogen Bond

We investigated five different amidated pentapeptides in water. The selection considers the displacement of the positive charge and hydrophobic residues along the peptide backbone. Table 1 lists the pentapeptides selected for this study.

**Figure 3:**
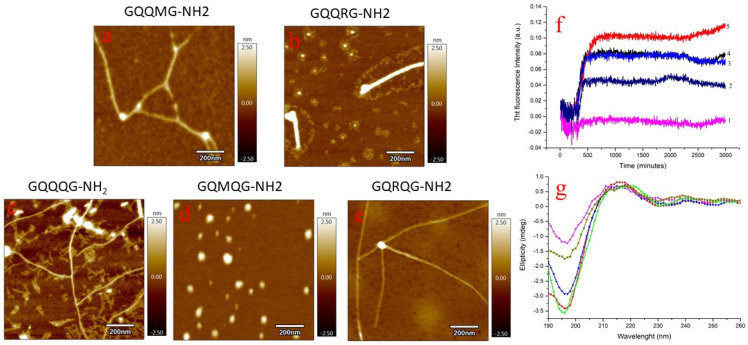
Liquid AFM measurements of GQQMG (**a**), GQQRG (**b**), GQQQG, (**c**) GQMQG (**d**), and GQRQG (**e**) peptides. AFM images were acquired after 500 min of incubation. Panel (**f**): ThT assay of GQMQG (1), GQRQG (2), GQQQG (3), GQQMG (4), and GQQRG (5) peptides. Panel (**g**): CD spectra acquired immediately after solubilization of the pentapeptides in water: GQMQG (green line), GQQMG (red line), GQQRG (blue line), GQRQG (dark yellow line), and GQQQG (magenta line). Peptide concentration was 10 µM, temperature 25 °C, pH = 7.4 and ionic strength 0.1 M in NaCl. In CD measurements, the ionic strength of the solutions was adjusted by NaF.

Figure 3f shows the results of the ThT kinetic assay for the investigated peptides. An increase in the fluorescence intensity associated with fibril formation was detected for all peptides except GQMQG. Since the ThT assay sometimes fails to detect fibril formation, atomic-force microscopy (AFM) measurements were also performed. GQQMG, GQQQG, and GQRQG peptides were shown to form fibrils, whereas GQQRG formed both globular and fibril structures. However, the GQMQG peptide only formed globular structures but no fibrils in agreement with the ThT assay results. All peptides assumed a random coil conformation in solution at zero incubation time, as circular dichroism (CD) spectra show (Figure 3g). After 500 min of incubation, attenuated total-reflectance infrared spectroscopy (ATR) spectra showed that all peptides assumed β-sheet conformation, as reported in Table 2, except GQRQG, which instead showed α-helix secondary structures. From the above data, arginine, a side chain with a positive charge, represses fibril formation, whereas a hydrophobic side chain promotes fibril formation.

### 2.3. Penta-Peptide GXXXG Interacts with Model Membrane Surface

**Figure 4:**
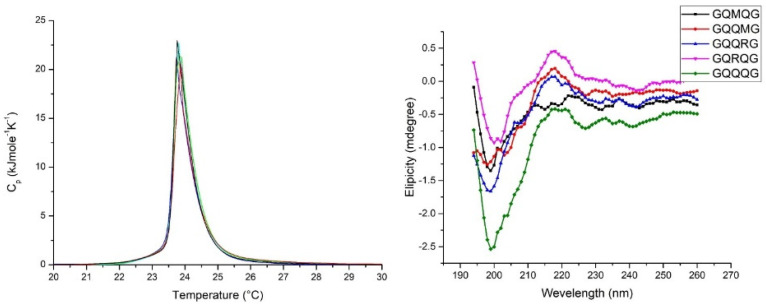
Left panel: DSC scan of DMPC SUV in the presence of penta-peptides. For the sake of clarity, the Cp profiles of GQRQG and GQMQG were not reported because of the overlap with the DMPC curve. Right panel: CD spectra of DMPC SUVs suspension in the presence of penta-peptides. SUV concentration, 1 mL/mL; penta-peptide concentration 40 µM; pH = 7.04 PBS buffer; ion strength 0.1.

Amyloidogenic polypeptides interact with model membranes by inserting into the bilayer and forming ion-channel-like pores. Differential scanning calorimetry (DSC) can detect the molecules’ insertion into the bilayer hydrophobic core by decreasing both the temperature and enthalpy associated with the gel–liquid crystal transition. If polypeptides remain on the bilayer surface, no change in temperature and enthalpy will be measured. In Figure 4, the left panel shows the Cp profile of the five investigated pentapeptides. No appreciable differences in temperature end enthalpy were observed with respect to pure DMPC, so we conclude that all peptides interact with the bilayer surface without any transfer into the bilayer core. According to the lipid chaperone hypothesis, pentapeptides cannot form the lipid–peptide complex with free phospholipid in the aqueous phase because there is only one hydrophobic side chain (methionine) in the pentapeptide sequence. Moreover, the peptide adsorbed on the membrane surface assumes a random coil conformation, as evidenced by CD measurements (right panel of Figure 4).

### 2.4. Penta-Peptide GXXXG in n-Octanol Mimic Hydrophobic Core of Bilayer Reveals C_α_―H···O Interaction

Quantum mechanics calculations revealed a peak centered at 2910 cm^−1^ assigned to the asymmetric vibration of the C-H interaction with C=O of the vicinal α-helix chain. To confirm the assignment, we investigated the formation of an interchain weak hydrogen bond between two vicinal pentapeptide molecules―we performed ATR measurements on all investigated peptides dissolved in *n*-octanol. The choice of *n*-octanol was based on avoiding the influence of the characteristic anisotropy of the bilayer, while maintaining the hydrophobicity (low relative dielectric constant of about 2.5), to prevent water interference with ATR measurements. Moreover, DSC measurements indicated that pentapeptides failed to penetrate the bilayer but remained adsorbed on the membrane surface.

**Figure 5:**
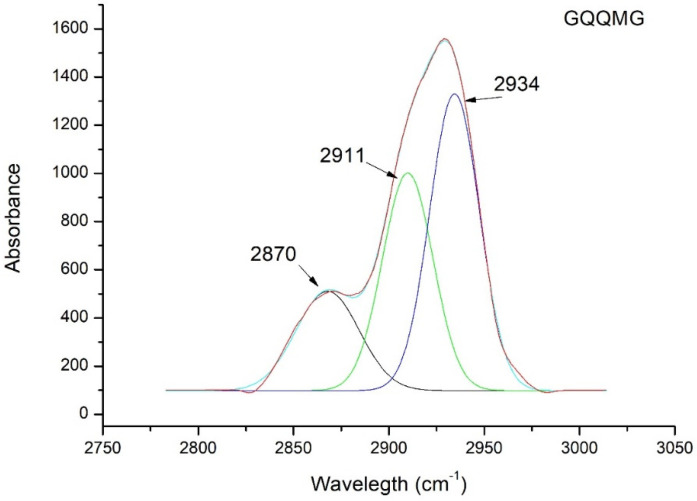
ATR deconvolution spectra of GQQMG pentapeptide in *n*-octanol.

Figure 5 shows the ATR spectrum deconvolution of GQQMG as an example. Table 3 reports the deconvolution spectra of all the peptides under study. According to QM simulations predicting a peak centered at 2910 cm^−1^, the ATR spectra showed a band very close to 2910 cm^−1^ for all the pentapeptides investigated, indicative of an intermolecular interaction between the glycine of a pentapeptide and an adjacent carbonyl group.

To best characterize the weak hydrogen bond interaction Cα―H···O between two vicinal α-helices, NMR measurements were performed on some of these pentapeptides.

Changes in the amide proton chemical shift with increasing temperature have been used to examine the strength of the hydrogen bond or intramolecular hydrogen bond formation [21]. The temperature coefficient of the amino acid residues for the corresponding peptides were determined by plotting the amide proton chemical shift as a function of temperature at intervals of 5K over the temperature range of 283 to 313 K. The results of temperature-dependent 1D ^1^H NMR spectra of the peptides tested here showed that all amide peaks exhibited a temperature-dependent displacement of their chemical shifts in the up-field direction. Distinct 1D peaks of these peptides were identified from 2D 1H-1H total correlation spectroscopy (TOCSY) spectra, and the values of temperature coefficients for the backbone amide protons ranged from −4 to −7 ppb/K in CD_3_OD. Additionally, the line shapes of the amide peaks remained more or less the same with increasing temperature suggesting a very slow exchange with the solvent molecules.

**Figure 6:**
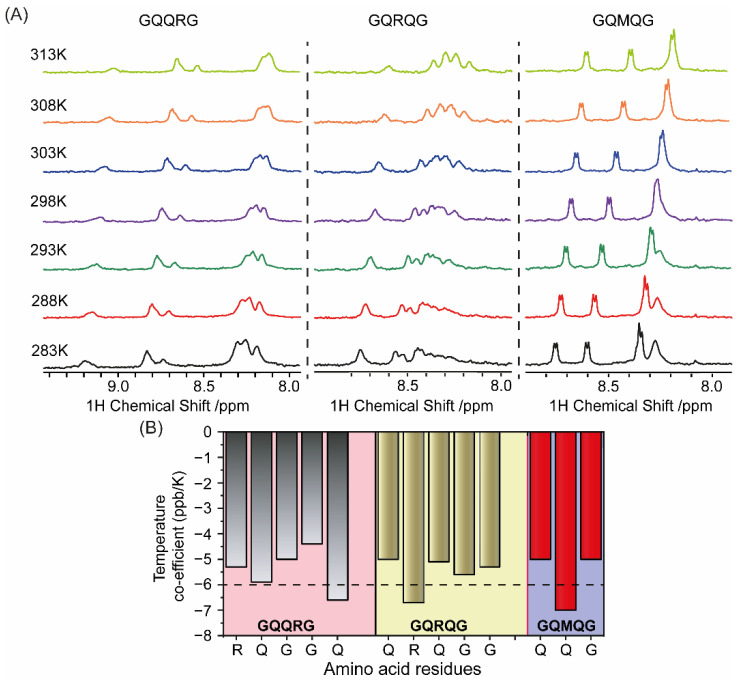
(**A**) Temperature-dependent NMR spectroscopy showing changes in the amide proton chemical shift of GQQRG, GQRQG, and GQMQG at different temperatures in CD3OD. (**B**) Temperature coefficients of the amino acid residues for the respective peptides from 1D ^1^H NMR were calculated and plotted as bar diagram (**B**).

The average amide proton temperature coefficients for the invariant glycine residues were −4.4 and −5 in GQQRG, −5.3 and −5.6 in GQRQG, and −5 in GQMQG. This can be attributed to some degree of conformational inclination resulting from the close contact of the glycine amide proton to Cα―H···O hydrogen bonding. However, slightly large negative values for glutamine in GQQRG (−6.6 ppb/K), arginine in GQRQG (−6.7 ppb/K), and glutamine in GQRQG (−7 ppb/K) were also observed. The side-chain amide protons also showed similar linear temperature dependence of chemical shifts. The temperature coefficients between −4 and −6 ppb/K and the slow exchange in CD3OD indicated that NH protons of the glycine residues for each peptide were fairly H-bonded (Figure 6A).

On the contrary, when measured in phosphate buffer (pH 7.4), the amide proton exchange for each peptide was very high, with a very high negative temperature coefficient (Figure S1). Changes in most side-chain amide protons’ chemical shifts were minimal, but the Cα―H chemical shifts were slightly downfield over this temperature range. Additionally, the amide proton peaks’ line shapes broadened rapidly with increasing temperature, suggestive of a very high exchange rate with the solvent water.

Thus, the four-residue separation between two glycine residues can induce hydrogen bonding under hydrophobic environments, irrespective of the residues in between this glycine. This could be an intrinsic property of the sequence, resulting from the absence of a side chain, with a concomitant lack of steric hindrance in the glycine residue.

## 3. Discussion

Aβ forms a stable complex with free phospholipids in the aqueous phase and then transfers to the bilayer. In the bilayer, it can form ion-channel-like pores with an inner diameter of a few nm but permeable to calcium ions. It has been proposed that these pores are stabilized by the GXXXG motif present in the C-terminus of Aβ capable of forming a hydrogen bond between a proximal α-helix chain. However, there are conflicting opinions in the literature on the stabilizing role of the GXXXG motif. Some authors suggest the stabilizing role of the GXXXG motif, while others bring opposite arguments, i.e., the GXXXG motif has no stabilizing role on ion-channel-like pores. In this investigation, pentapeptides having the GXXXG motif were synthesized and investigated in water and membranes.

In water, all peptides studied at zero time were in random coil configuration (Figure 3g) and, after 500 min, formed fibrils except for the GQMQG sequence as shown by the ThT assay and AFM measurements. ATR measurements (Table 2) on fibril evidenced β–sheet structures in all peptides, excluding the GQRQG sequence where α-helix was detected by ATR (Table 2).

Previous molecular dynamics data show that Aβ (1-40) in the membrane is stable as an α-helix and forms aggregates [19]. These aggregates consist of ion channels allowing the passage of water and calcium ions. The single ion channel is composed of four Aβ (1-40) molecules in α-helix conformation, with the C=O and an H-N groups in two proximal chains falling within a distance of 3.2 Å, sufficient to form a hydrogen bond (Figure 1). To verify whether the hydrogen bond was formed, quantum mechanics calculations were performed, and the infrared spectrum of the atoms involved in the interaction was simulated. The simulated infrared spectrum showed the presence of a peak centered at 2910 cm^−1^ attributed to the formation of a hydrogen bond between the C=O of Gly7 and the NH of Asp23.

To support the simulated infrared spectra (Figure 2), experiments of pentapeptides in the presence of model membranes were done. DSC measurements clearly show that the investigated peptides do not insert into the bilayer but remain adsorbed on the membrane surface in β-sheet conformation (Figure 4). This behavior can be explained by considering the lipid-chaperone model, which suggests that amyloidogenic proteins are transported into the hydrophobic membrane core if a stable complex is formed between the polypeptide and free lipids in the aqueous phase. This complex can insert into the membrane due to higher hydrophobicity than the polypeptide alone. However, the polypeptides investigated here were not hydrophobic enough to form a stable complex and therefore did not enter the membrane but remained adsorbed on its surface. To overcome the failure to insert the polypeptide into the bilayer, we used n-octanol to solubilize the polypeptides in a solvent with a low dielectric constant and avoid the presence of water that interferes with ATR measurements. Figure 5 and Table 3 evidence the presence of a peak close to 2910 cm^−1^ in agreement with the simulated infrared spectra. Further confirmation comes from NMR measurements that agree with the ATR data.

## 4. Materials and Methods

### 4.1. Chemicals

*N*-Fluorenylmethoxycarbonyl (Fmoc)-protected amino-acids, NovaSyn-TGR resin (loading 0.18 mM/g, 0.33 mM scale synthesis), and NovaSyn-TGRA resin (loading 0.18 mM/g, 0.33 mM scale synthesis) were purchased by Merck (Darmstadt, Germany); *N*,*N*-diisopropyl-ethylamine (DIEA), *N*,*N*-dimethylformamide (DMF, peptide synthesis grade), piperidine, dimethylformamide (DMF), *N*-hydroxybenzotriazole (HOBt), 2-(1-H-benzotriazole-1-yl)-1,1,3,3-tetramethyluronium tetrafluoroborate (TBTU), triisopropylsilane (TIS), cloroform, and trifluoroacetic acid (TFA), were purchased from Sigma-Aldrich (Munich, Germany). Peptide solution was prepared by dissolving lyophilized peptide in water. 1,2-dimyristoyl-sn-glycero-3-phosphocholine (DMPC) was purchased from Avanti Polar Lipids (USA). Ultrapure Milli-Q water (resistivity > 18 MΩ·cm^−1^) was used for all experiments.

### 4.2. Penta-Peptide Synthesis

A microwave-assisted solid-phase-peptide-synthesis (MW SPPS) approach was used to achieve the following peptide sequences on a Biotage^®^ Initiator + SP (Biotage, Uppsala, Sweden). Pentapeptides were prepared with a free and amidated C-terminal carboxyl group moiety by means of the use of the corresponding resins: TGA and TGR. Amino acids were activated through the coupling TBTU/HOBt procedure. Briefly, the coupling reaction was carried out at room temperature, by using amino acid excess (5-fold vs. the resin) and HOBt/TBTU/DIPEA (5 equivalent) in DMF, under mixing for 10 min. Fmoc deprotection steps were performed at room temperature using 20% of piperidine in DMF for 15 min. The resin was washed with dichloromethane and dried on a synthesizer.

The next steps are analogous to those previously reported. Briefly, the peptides were cleaved off from their respective resins and simultaneously deprotected by treatment with a mixture of TFA/ TIS/H2O (95/2.5/2.5 *v*/*v*) for 1.5 h at room temperature. Every solution containing the free peptide was filtered off from the resin and concentrated in vacuo at 30 °C. The peptide was precipitated with cold diethyl ether. The precipitate was then filtered, dried under vacuum, re-dissolved in water, and lyophilized. Analytical rp-HPLC analyses were performed using a Waters 1525 instrument, equipped with a Waters 2996 photodiode array detector. The peptide samples were analyzed using gradient elution with solvent A (0.1% TFA in water) and B (0.1% TFA in acetonitrile) on a Vydac C18 250 4.6 mm (300 Å pore size, 5 μm particle size) column, run at a flow rate of 1 mL/min. Linear gradient 0 to 5% B over 25 min.

All samples were analyzed by ESI-MS to verify the success of the syntheses. GQQQG-NH2: (Rt = 8.2 min). Mass calculated for C19H33N9O8, M = 515.52, ESI–MS (obsd. *m*/*z*: (M+H)+ 516.5); GQQQG-OH: (Rt = 9.0 min). Mass calculated for C19H32N8O9, M = 516.51, ESI–MS (Obsd. *m*/*z*: (M+H)+ 517.6); GQQRG-NH2: (Rt = 7.5 min). Mass calculated for C N-Fluorenylmethoxycarbonyl (Fmoc)-protected amino-acids. NovaSyn-TGR resin (loading 0.18 mM/g, 0.33 mM scale synthesis) resin and NovaSyn-TGRA resin (loading 0.18 mM/g, 0.33 mM scale synthesis) resin were purchased from Merck (Darmstadt, Germany); *N*,*N*-diisopropyl-ethylamine (DIEA), *N*,*N*-dimethylformamide (DMF, peptide synthesis grade), piperidine, dimethylformamide (DMF), N-hydroxybenzotriazole (HOBt), 2-(1-H-benzotriazole-1-yl)-1,1,3,3-tetramethyluronium tetrafluoroborate (TBTU), triisopropylsilane (TIS), and trifluoroacetic acid (TFA) were purchased from Sigma-Aldrich (Munich, Germany). Peptide solution was prepared by dissolving lyophilized peptide in water. Ultrapure Milli-Q water (resistivity > 18 MΩ·cm^−1^) was used for all experiments. 20H37N11O7, M = 543.58, ESI–MS (Obsd. *m*/*z*: (M+H)+ 544.7); GQRQG-NH2: (Rt = 7.5 min). Mass calculated for C20H37N11O7, M = 543.58, ESI–MS (Obsd. *m*/*z*: (M+H)+ 544.6); GQQMG-NH2: (Rt = 9.2 min). Mass calculated for C19H34N8O7S, M = 518.59, ESI–MS (Obsd. *m*/*z*: (M+H)+ 519.6); GQMQG-NH2: (Rt = 9.0 min). Mass calculated for C19H34N8O7S, M = 518.59, ESI–MS (Obsd. *m*/*z*: (M+H)+ 519.6).

### 4.3. Quantum Mechanics

The two α-helix chains selected from previous MD simulations [19] are characterized by the following sequences:

c_1_ NH2-Ala^1^-Glu^2^-Asp^3^-Val^4^-Gly^5^-Ser^6^-Asn^7^-Lys^8^-Gly^9^

c_2_ NH2-Asp^1^-Val^2^-Gly^3^-Ser^4^-Asn^5^-Lys^6^-Gly^7^-Ala^8^-Ile^9^

The selected structure was fully optimized using the Perdew, Burke, Ernzerhof functional [22], and harmonic vibrational frequencies, for the equilibrium structure obtained, were evaluated. The calculations were performed by using the 6-31G basis set augmented with polarization and diffuse functions (6-31+G**), Wiberg bond order [23], which is a measure of electron population overlap between two atoms, was evaluated as follows:

(1) $$I_{AB}=\underset{\mu \in A}{\sum }\underset{\nu \in B}{\sum }P_{\mu \nu }^{2}$$

where *μ* are the atomic orbitals on atom A, *ν* are the orbitals on atom B, and *P^2^_μν_* is the correlated density matrix element. All the calculations were performed with the Gaussian 16 software [24]. The NBO [25] procedure is called by using the keywords pop = nbored and is followed, after the geometry specification, by: $NBO BNDIDX $END.

### 4.4. NMR Measurements

Temperature-dependent 1D NMR spectra were recorded either on a Bruker Avance III 500 MHz NMR spectrometer equipped with a 5 mm SMART probe or on a Bruker Avance III 700 MHz NMR spectrometer, equipped with a RT probe. Peptide samples were prepared either in methanol-d4 (CD_3_OD) or in phosphate buffer (20 mM phosphate, pH 7.4), added with 10% D_2_O. To the sample containing 600 µL final volume, trimethylsilylpropionic acid (TSP) was added as a reference for all the NMR experiments. For each peptide, 1D NMR measurements were recorded at different temperatures (10 °C, 15 °C, 20 °C, 25 °C, 30 °C, 35 °C, and 40 °C), keeping the same parameters in each experiment. Two-dimensional ^1^H-^1^H total correlation spectroscopy (2D TOCSY) spectra were recorded for the peptides at 15 °C, with a mixing time of 80 ms and with 24 scans.

### 4.5. AFM Measurements

AFM analysis was performed on a Cypher microscope (Oxford Instruments, Santa Barbara, CA, USA) equipped with a scanner operating at XY scan range of 30/40 μm (closed/open loop). To prepare the samples, a 10 μL volume of peptide solution was dispensed on freshly cleaved muscovite mica (Ted Pella, Inc., Redding, CA, USA). After 5 min of incubation, samples were dried under a gentle N_2_ flow. Scan images in random areas of the samples were acquired in AC mode imaging in air by using Si cantilevers (OMCL-AC240TS, ~70 kHz, 2 N/m, Olympus, Tokyo, Japan). AFM images of height were analyzed using the free tool in the MFP-3DTM offline analysis software.

### 4.6. ThT Assay

ThT measurements. The kinetics of fiber formation was measured using Thioflavin T (ThT) assay. Samples were prepared by diluting in phosphate buffer 10 mM, 100 mM NaCl, pH 7.4 the opportune amount of pentapeptide stock solution to reach 10 μM concentration. Thioflavin T was then added to a final concentration of 20 µM. Experiments were carried out in Corning 96-well non-binding surface plates. Time traces were recorded using a Varioskan (ThermoFisher, Waltham, MA, USA) plate reader using a λ_ecc_ of 440 nm and a λ_em_ of 485 nm at 37 °C, shaking the samples for 10 s before each read. All ThT curves represent the average of three experiments.

### 4.7. Circular Dichroism

CD spectroscopy was used to assess the change in pentapeptide secondary structure in solution. Spectra were recorded with a (JASCO PTC 348) spectropolarimeter at 25° C. Peptide solutions of 5 µM were prepared in water and measured using a quartz cuvette with a path length of 1 cm, from 250 to 190 nm. The spectra of each peptide were taken at two different intervals: 0 and 48 h. All the spectra were blank corrected by subtraction of the background experiment.

### 4.8. LUV Preparation

We used large unilamellar vesicles (LUVs) composed by a zwitterionic lipids (DMPC). Model membranes were prepared as described elsewhere [26]. Briefly, appropriate aliquots of lipid stock solutions in chloroform were dried by using a stream of dry nitrogen and evaporated overnight under high vacuum to dryness in a round-bottomed flask. Initially multilamellar vesicles (MLVs) were obtained by hydrating the lipid film with an appropriate amount of buffer (phosphate buffer 10 mM, 100 mM NaCl, pH 7.4) and dispersing by vigorous stirring. MLVs were then extruded 21 times to obtain LUVs.

### 4.9. DSC Experiments

DSC runs of 1 mg/mL DMPC LUVs in the presence of 40 μM of each pentapeptide were carried out on a Nano-DSC (TA Instruments, Inc., New Castle, DE) apparatus. Lipid samples were degassed by vacuum and then heated from 10 to 40 °C at a scan rate of 1 °C min^−1^. An extra external pressure of about 3 atm was applied on the solution to prevent the formation of bubbles during heating. The phosphate buffer solution 10 mM, 100 mM NaCl, pH 7.4 was used as reference. Heat-capacity curves (Cp) were obtained by subtracting the buffer–buffer baseline from raw DSC data [27]. All DSC runs were performed immediately after the preparation of samples.

### 4.10. ATR-FTIR Spectroscopy

The change in pentapeptide secondary structure was evaluated by attenuated total reflectance spectroscopy. Spectra of the synthetic peptides were acquired using an Infrared Bruker Tensor 37 spectrometer. Sample solutions with a concentration of 5 mM were prepared in water and deposited (10 μL) on a clean microscope glass slide. Subsequently, the latter were dried in a soft nitrogen stream and then scanned from 4000 to 400 cm^−1^ using a spectral resolution of 1 cm^−1^. The structural changes were monitored following the shifts of the amide I band (1700–1600 cm^−1^), which corresponds almost exclusively to the C=O stretch vibrations of the peptide bonds.

## 5. Conclusions

Previously, some of us investigated the role of the GXXXG sequence in Aβ (1-40) toxicity [19]. Some papers in the literature report contradictory data on the ability of the GXXXG sequence to form a hydrogen bond (Cα―H···O) that stabilizes Aβ pores in membranes. In the present work, we used simulation and experimental techniques to characterize the physicochemical behavior of the GXXXG sequence in water and within the membrane and its ability to form intermolecular hydrogen bonds between two adjacent helical peptides of the ion-channel like pore.

We started from our previous MD simulations to select the coordinates of two adjacent α-helix peptides of the Aβ transmembrane pore. These coordinates were thus used to perform QM calculations to evaluate the IR spectrum. The simulated spectrum has evidenced the formation of a weak hydrogen bond between the two selected chains. An experimental investigation was also designed to evaluate the impact of the external aqueous or lipid environments on the ability of the GXXXG sequence to form hydrogen bonds. Penta-peptides containing the GXXXG sequence formed fibrils in water but lacked the Cα―H···O hydrogen bond. In the presence of LUVs, pentapeptides remained adsorbed on the membrane surface without forming pores. This behavior was attributed to the low hydrophobicity of these pentapeptides which limited their ability to penetrate the membrane core [7,9]. To overcome this limitation, the peptides were instead solubilized in *n*-octanol as a mimic of the membrane environment. NMR and ATR measurements of the solution containing pentapeptide dissolved in n-octanol confirmed the presence of the Cα―H···O hydrogen bond.

In conclusion, our data confirmed the formation of a Cα―H···O hydrogen bond, that is GXXXG-sequence-independent, and which can stabilize ion-channel-like pores in the membrane.

## Figures and Tables

**Table 1:** Investigated penta-peptides. **G**, Gly, glycine, hydrophobic; **Q**, Gln, glutamine, neutral hydrophilic; **R**, Arg, arginine, positively charged; **M**, Met, methionine, hydrophobic.

Penta-Peptide
**GQQQG-NH_2_**
**GQQRG-NH_2_**
**GQRQG-NH_2_**
**GQQMG-NH_2_**
**GQMQG-NH_2_**

**Table 2:** Secondary structure assignment of penta-peptides using ATR spectroscopy measurements. Spectra were acquired after 500 min of incubation. Frequency assignment from reference [20].

Peptide	AssignmentAssignment	Frequency (cm^−1^)
**GQQRG-NH_2_**	β-sheet	1624
**GQRQG-NH_2_**	α-helix	1656
**GQQMG-NH_2_**	β-sheet	1633
**GQMQG-NH_2_**	β-sheet	1627
**GQQQG-NH_2_**	β-sheet	1624

**Table 3:** Deconvolution of ATR peak of five pentapeptides to detect the C^α^―H···O hydrogen bond. The frequency calculated from quantum mechanics simulations was 2910 cm^−1^.

Peptide	Frequency (cm^−1^)
**GQQQG**	2908
**GQQRG**	2910
**GQRQG**	2907
**GQQMG**	2910
**GQMQG**	2907

## Data Availability

QM coordinate are available at GitHub.

## Supplementary Materials

The following supporting information can be downloaded at: https://www.mdpi.com/article/10.3390/ijms24032192/s1.
