# Supplementary material for: GxxxG Motif Stabilize Ion-Channel like Pores through Cα―H···O Interaction in Aβ (1-40)
Source: Int J Mol Sci. 2023 Jan 22;24(3):2192. doi: 10.3390/ijms24032192 (PMC9917128; doi:10.3390/ijms24032192)
Supplement: Supplementary file 1 [file ijms-24-02192-s001.zip › ijms-2145727-supplementary.pdf]

Supporting Information

GxxxG motif stabilize ion-channel like pores through Cα—H···O interaction in Aβ (1-40)

Carola Rando[1], Giuseppe Grasso[1], Dibakar Sarkar[2], Michele F.M. Sciacca[3], Lorena M. Cucci[1], Alessia Cosentino[1], Giuseppe Forte[4], Martina Pannuzzo[5], Cristina Satriano[1], Anirbarn Bhunia[2] and Carmelo La Rosa[1,*]

[1]Dipartimento di Scienze Chimiche, Università degli Studi di Catania, Viale A. Doria 6, 95125 Catania, Italy

[2]Department of Biophysics Bose Institute, Unified Academic Campus, Bidhan Nagar, EN 80, Kolkata 700 091, India

[3]Consiglio Nazionale delle Ricerche, Istituto di Cristallografia, Catania, Italy

[4]Dipartimento di Scienze del Farmaco e della Salute, Università degli Studi di Catania, Viale A. Doria 6, 95125 Catania, Italy

[5]Laboratory of Nanotechnology for Precision Medicine, Fondazione Istituto Italiano di Tecnologia, Via Morego 30, Genoa 16163, Italy

* Correspondence: Carmelo La Rosa

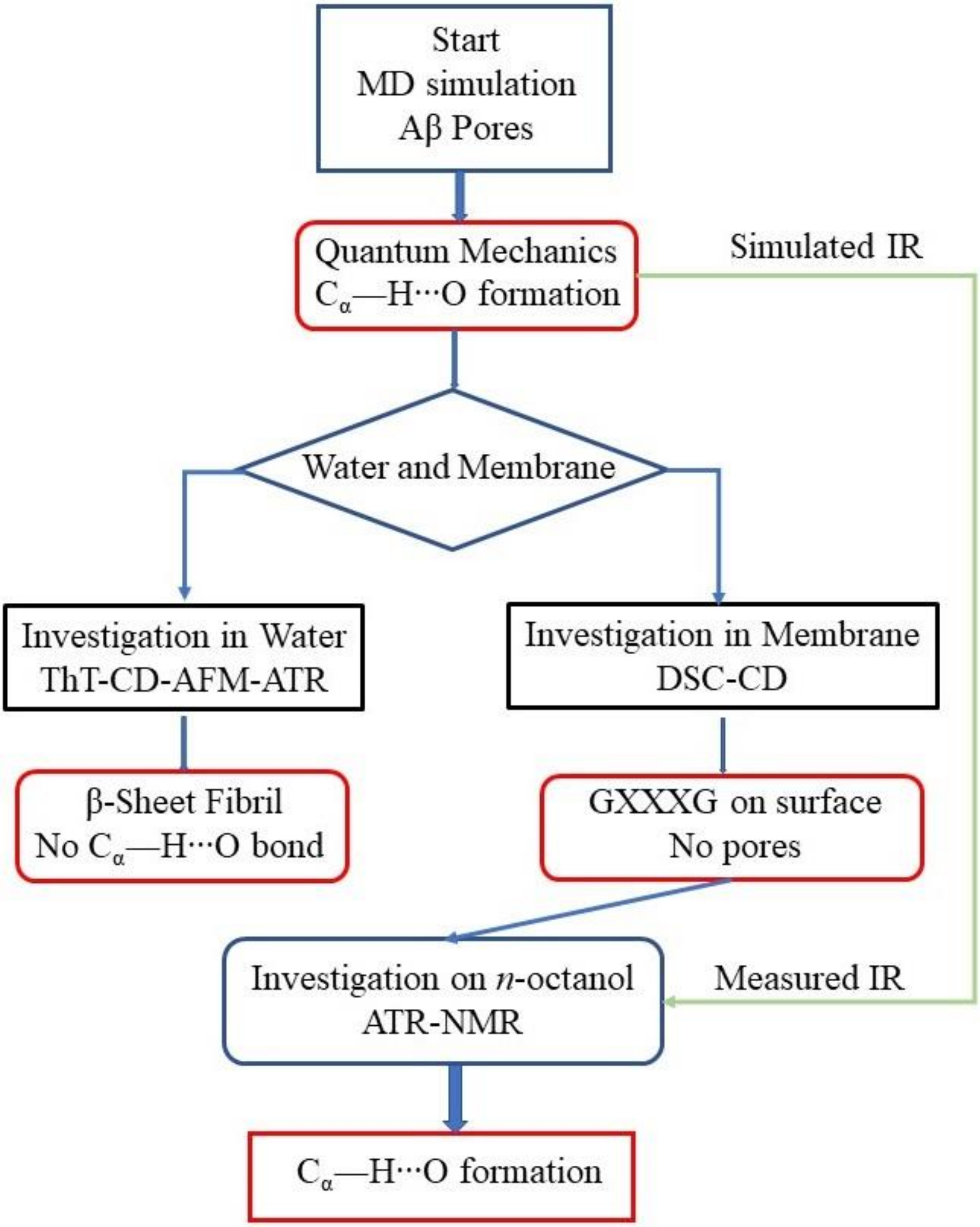

Figure S1. Workflow of present work. In red the conclusions.
